# Supplementary material for: Shared features and reciprocal complementation of the Chlamydomonas and Arabidopsis microbiota
Source: Nat Commun. 2022 Jan 20;13:406. doi: 10.1038/s41467-022-28055-8 (PMC8776852; doi:10.1038/s41467-022-28055-8)
Supplement: Supplementary file 1 — Supplementary Information [file 41467_2022_28055_MOESM1_ESM.pdf]

Shared features and reciprocal complementation of the *Chlamydomonas* and *Arabidopsis* microbiota

Paloma Durán[1,2,†], José Flores-Uribe[1,†], Kathrin Wippel[1], Pengfan Zhang[1], Rui Guan[1], Barbara Melkonian[1], Michael Melkonian[1], Ruben Garrido-Oter[1,2,+]

Correspondence to: garridoo@mpipz.mpg.de

[1] Department of Plant-Microbe Interactions, Max Planck Institute for Plant Breeding Research, 50829 Cologne, Germany.

[2] Cluster of Excellence on Plant Sciences, 40225 Düsseldorf, Germany.

[†] Co-first authors

[+] Correspondence to: garridoo@mpipz.mpg.de

This PDF file includes the following Supplementary Information:

Supplementary Figures 1-9.

Supplementary Tables 1-2.

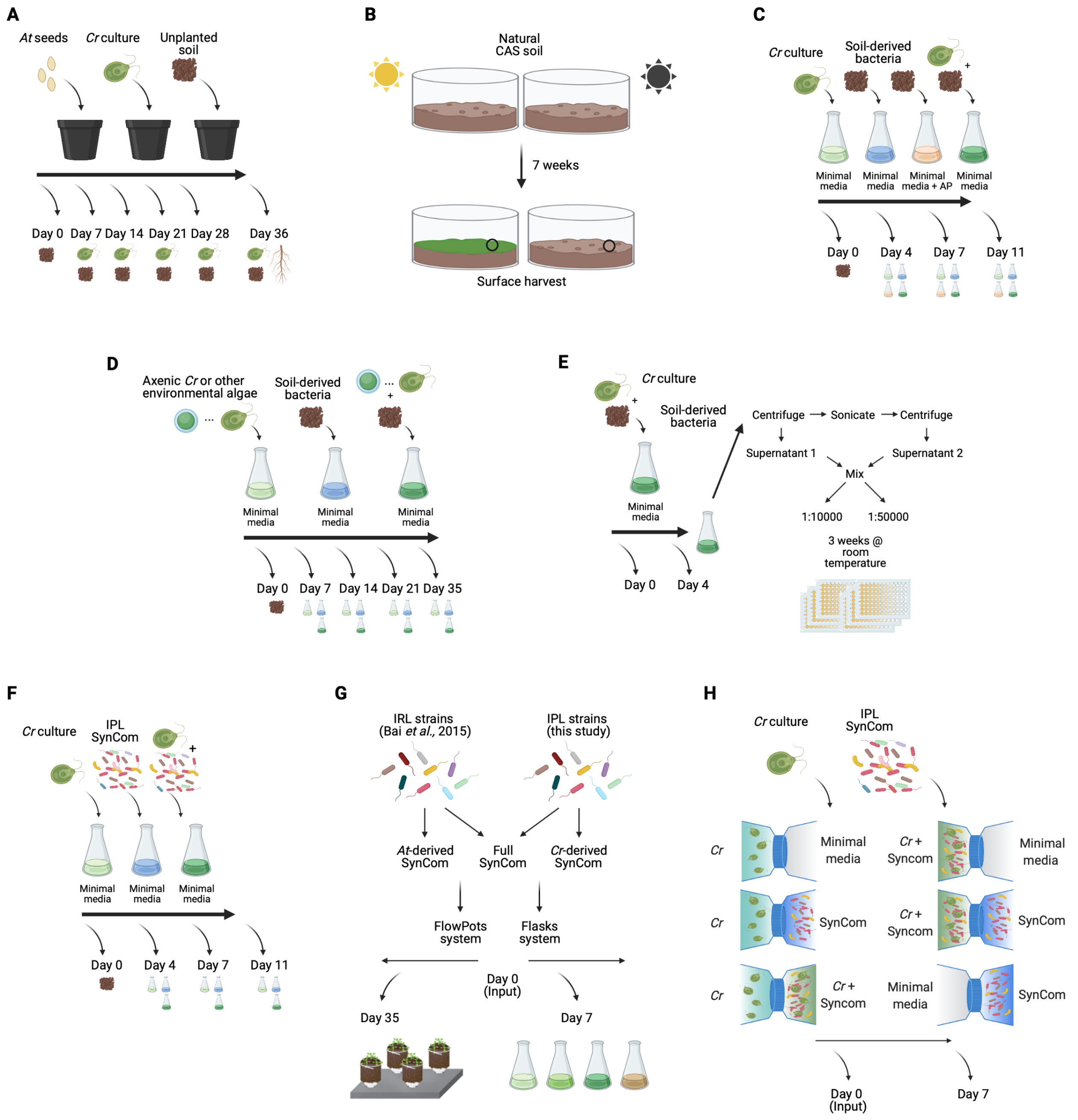

Supplementary Figure 1: Schematic description of the experimental approaches employed in this study (caption on next page).

Supplementary Figure 1 | Schematic description of the experimental approaches employed in this study (figure on previous page).

(**a**) Greenhouse experiment. Pots containing CAS natural soil were either sown with *At* seeds, inoculated with *Cr* cultures or mock-treated. Samples were taken over time for bacterial community profiling using *16S* rRNA amplicon sequencing. (**b**) Soil native algae community experiment. CAS soil was placed in gnotobiotic boxes and inside growth chambers with a day / night cycle or in the dark. After 7 weeks, samples from the initial soil, and soil surfaces of lighted and dark boxes were subjected to *16S* and *18S* rRNA profiling for bacterial and eukaryotic community analysis. (**c**) Mesocosm experiments. In different liquid media (minimal media or AP-containing media), *Cr* cultures were co-incubated with soil-derived bacteria. Samples were taken over time for bacterial community profiling and assessment of *Cr* growth. (**d**) Environmental subaerial algal strains experiment. Using liquid medium, axenic algal cultures were co-inoculated with soil-derived bacteria. Samples for bacterial community profiling using *16S* rRNA were taken over time. (**e**) Protocol for the establishment of the *Cr*-IPL bacterial culture collection generation. *Cr* cultures were co-incubated with soil-derived bacteria for 7 days and fractionated to enrich for phycosphere bacteria. This phycosphere fraction was then diluted and incubated for several weeks in 96-well plates. Subsequently, bacterial cultures were subjected to *16S* rRNA amplicon profiling for further analysis (**f**) SynCom reconstitution experiment. From the core culture *Cr*-SPHERE collection, 26 representative strains were selected, pooled together and co-incubated with *Cr* in minimal media for 7 days. (**g**) Cross-inoculation experiment. One bacterial strain per shared bacterial family from the *At*-SPHERE and *Cr*-SPHERE were selected and assembled into root or phycosphere SynComs. These SynComs were cross-inoculated in *At* and *Cr* in two gnotobiotic systems (i.e., soil-based FlowPots and liquid-based flasks), individually or in a mixed community. Root and phycosphere samples were harvested after 5 weeks of co-incubation for community profiling and assessment of *Cr* growth. (**h**) Split co-cultivation system. Co-cultivation chambers separated by a 0.22 µm filter were used to inoculate *Cr*, the phycosphere SynCom alone or both together in different combinations. Samples were harvested after 7 days for *16S* rRNA bacterial community profiling and *Cr* growth measurements. Elements of this figure were created with biorender.com .

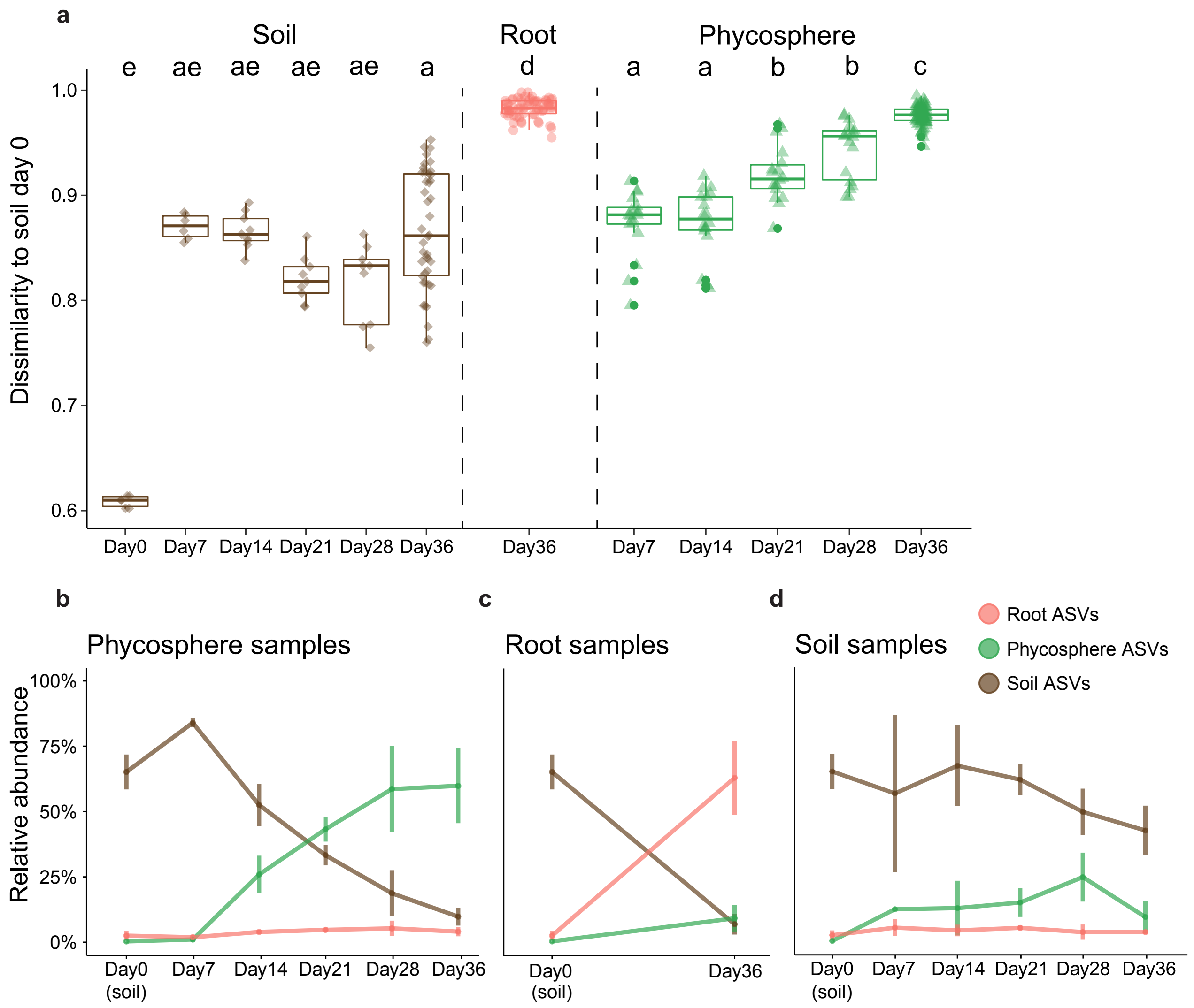

Supplementary Figure 2 | Culture-independent analysis of phycosphere- and root-associated communities in a natural soil.

(**a**) ASV-level analysis of Bray-Curtis dissimilarities of bacterial communities from phycosphere, root and soil samples (*n*=95), compared to the initial soil input (day 0, n=3). Boxplots are color-coded depending on the fraction. Significant differences are marked with different letters (two-sided Kruskal-Wallis test, followed by a Dunn's *post hoc*, with Bonferroni correction; $P<0.05$). (**b-d**) Dynamics of relative abundances of ASVs enriched in phycosphere (*n*=48, **b**), root (*n*=19, **c**) or soil samples (*n*=28, **d**) over time, compared to initial soil input (day 0; Wilcoxon test; $P<0.05$). Number of replicates indicated in parenthesis belong the technical replicates across 3 biological replicates. Curves are color-coded depending on the fraction indicated. Corresponds to experiment A (**Supplementary Fig. 1a** and **Table1**).

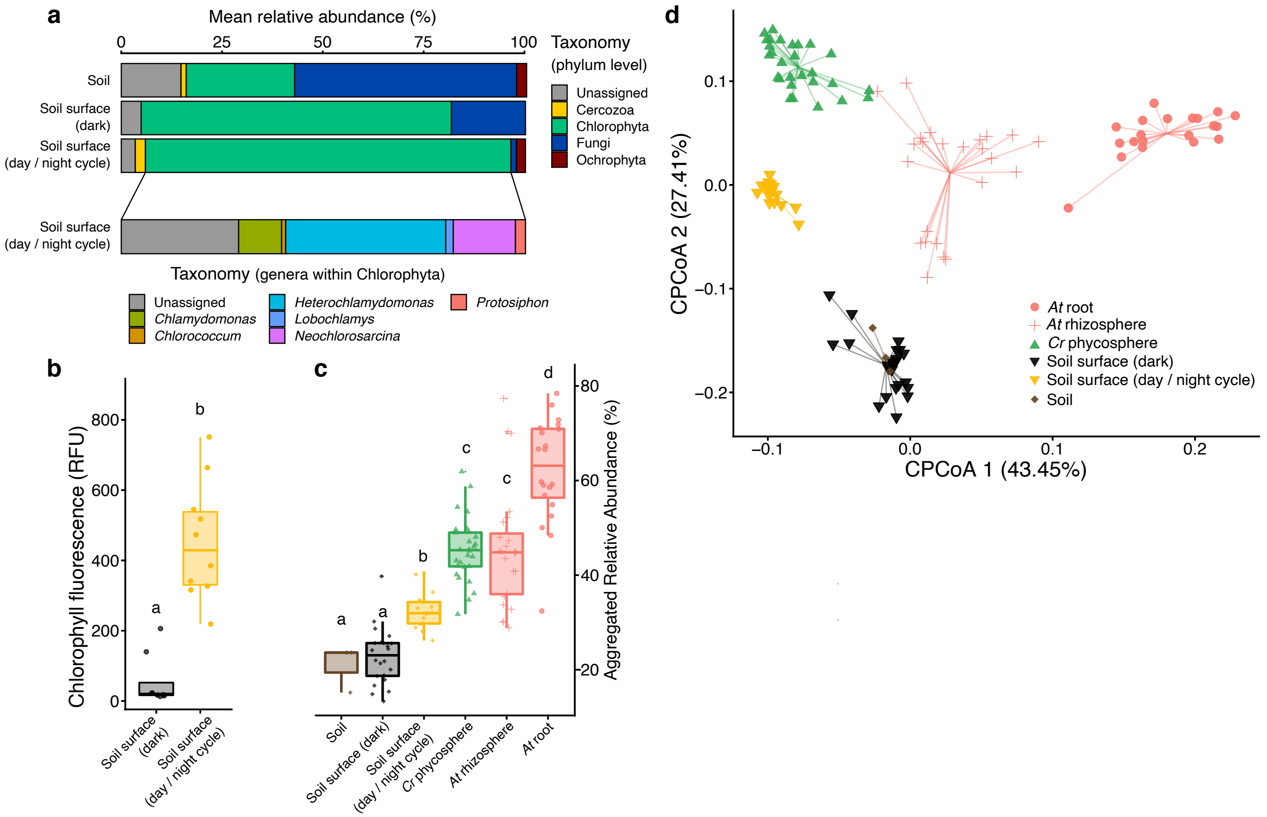

Supplementary Figure 3 | Growth of native microalgae in a natural soil induces shifts in bacterial community structure. (**a**) Taxonomic composition of samples from natural soil based on *18S* rRNA gene profiles at the beginning of the experiment and after 7 weeks of incubation in growth chambers under day / night or dark conditions. The composition of the Chlorophyta from the light-treated samples at the genus level is shown as an inset. (**b**) Chlorophyll content (relative fluorescence) of soil surface samples under light (n=10), and dark (n=8) conditions. (**c**) Aggregated relative abundance of core root and phycosphere bacterial taxa in the different fractions (n=114). A two-sided Kruskal-Wallis test followed by a Dunn's *post hoc* with Bonferroni correction was used to assess significant differences among groups (P<0.05). (**d**) Beta diversity (Bray-Curtis dissimilarities) analysis of soil surface bacterial *16S* rRNA gene community profiles (n=114) at the ASV level shown as CPCoA (19.6% of the variance; P<0.001). Corresponds to experiment B (**Supplementary Fig. 1b** and **Supplementary Table 1**). *Cr* phycosphere and *At* root and rhizosphere samples were obtained from the previous greenhouse experiment (exp. A; **Fig. 1b-c**, **Supplementary Fig. 1a** and **Supplementary Table 1**).

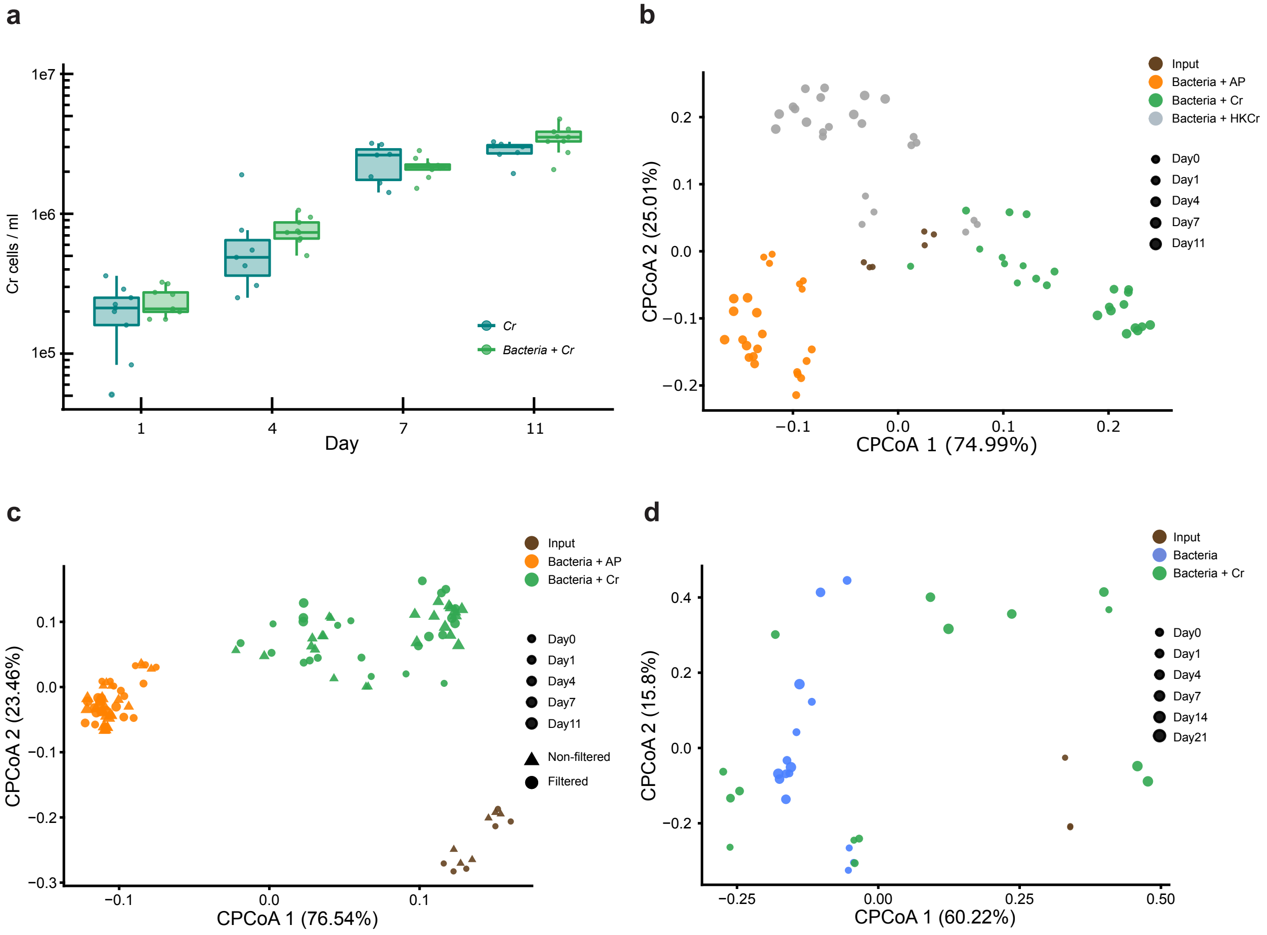

Supplementary Figure 4 | Analysis of phycosphere communities in mesocosm experiments.

(**a**) Cell counts of axenic *Cr* cultures (*n*=32, dark green) and of *Cr* co-inoculated with soil-derived bacteria (*n*=32, light green) over time. No significant differences were found between growth conditions at each time point (two-sided Wilcoxon test). (**b**) PCoA of Bray-Curtis dissimilarities constrained by the experimental condition (17.5% of variance; *P*<0.001) of soil-derived bacterial communities co-inoculated with AP (*n*=24), live *Cr* cultures (*n*=24) or heat-killed *Cr* (*n*=24). (**c**) PCoA of Bray-Curtis dissimilarities constrained by the experimental condition (17.5% of variance; *P*<0.001) of filtered (*n*=54, circles) or non-filtered (*n*=54, triangles) bacterial communities derived from soil and co-inoculated with AP or *Cr* cultures. No significant separation of samples was found based on filtration treatment (0.73% of variance). (**d**) PCoA of Bray-Curtis dissimilarities constrained by experimental condition and time point of (40.1% of variance; *P*<0.005) of soil-derived bacterial communities either co-inoculated with *Cr* cultures (*n*=15) or alone in minimal media, under day/night light conditions (*n*=15). All beta-diversity analyses were performed at the ASV level. Complements experiment C (**Supplementary Fig. 1c** and **Supplementary Table 1**).

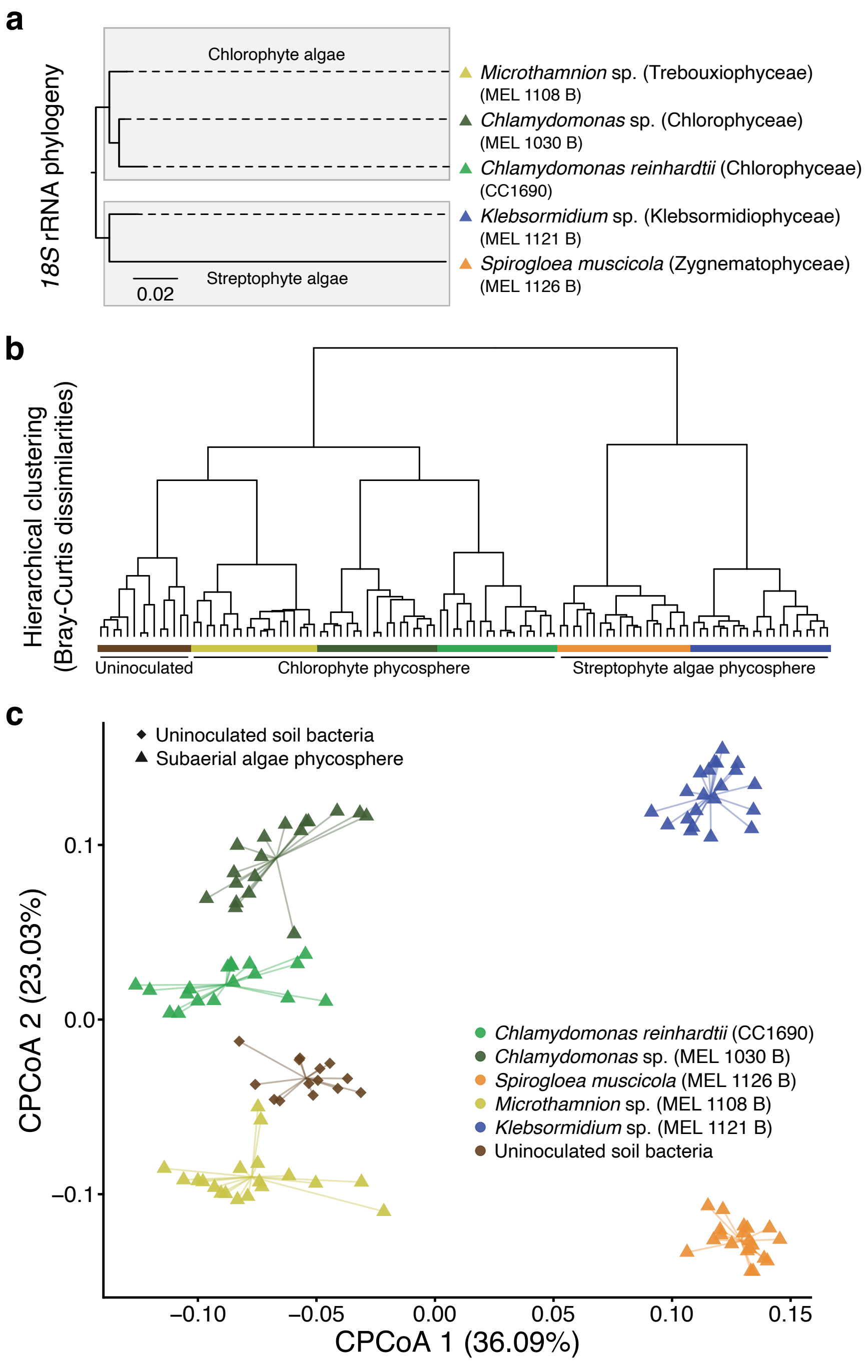

Supplementary Figure 5 | Phycosphere bacterial community structure depends on the host taxonomy. (**a**) Maximum Likelihood phylogeny of *18S* rRNA sequences of different representatives of Chlorophyte and Streptophyte microalgae. (**b-c**) Beta diversity analysis (Bray-Curtis dissimilarities) of phycosphere bacterial *16S* rRNA gene community profiles at the ASV level obtained from different microalgae (n=110). Samples were compared using hierarchical clustering (**b**), and CPCoA (16.9% of the variance; P<0.001) (**c**). Corresponds to experiment D (**Supplementary Fig. 1d** and **Supplementary Table 1**).

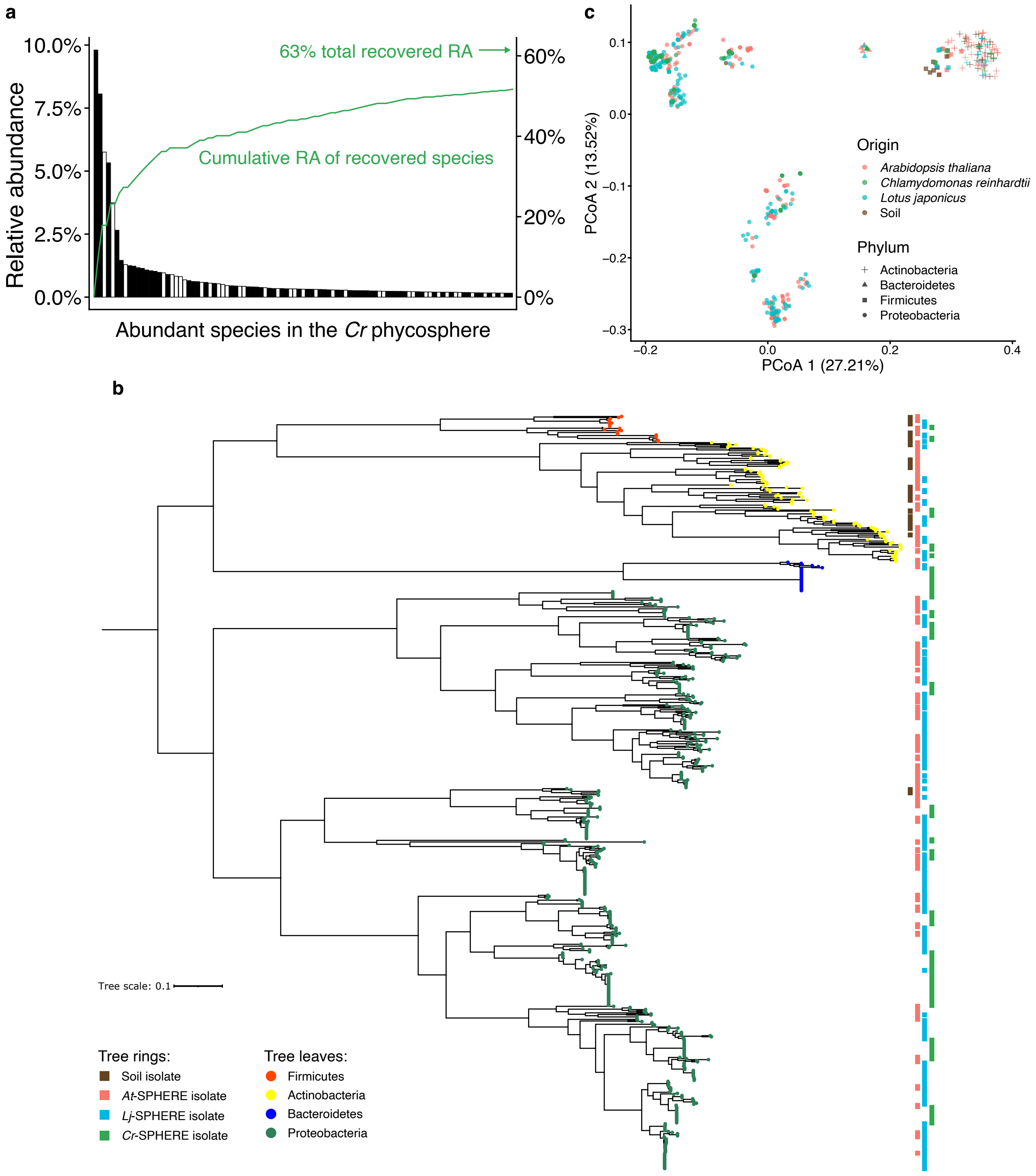

Supplementary Figure 6 | Characterization of the IPL and *Cr*-SPHERE culture collection (caption on next page).

Supplementary Figure 6 | Characterization of the IPL and *Cr*-SPHERE culture collection (figure on previous page).

(**a**) Bar plot showing the relative abundance of the top 100 most abundant OTUs found in culture-independent phycospheres (experiment A) indicating whether a representative exemplar was recovered in the IPL bacterial library (62%). The cumulative relative abundance curve and arrow represent total contribution of recovered OTUs to the culture-independent phycosphere communities (63% of the entire community). (**b**) Whole-genome phylogenetic tree of bacterial strains derived from soil or the *At*-, *Lj*-, *Cr*-SPHERE culture collections based on a concatenation of single copy phylogenetic markers known as AMPHORA genes. Tree leaves are colored by taxonomic affiliation, while colored boxes represent the origin of each genome. (**c**) PCoA of functional distances of genomes from bacterial isolates of the *Chlamydomonas* (Cr-SPHERE), *Arabidopsis* (At-SPHERE), and *Lotus* (Lj-SPHERE) bacterial culture collections.

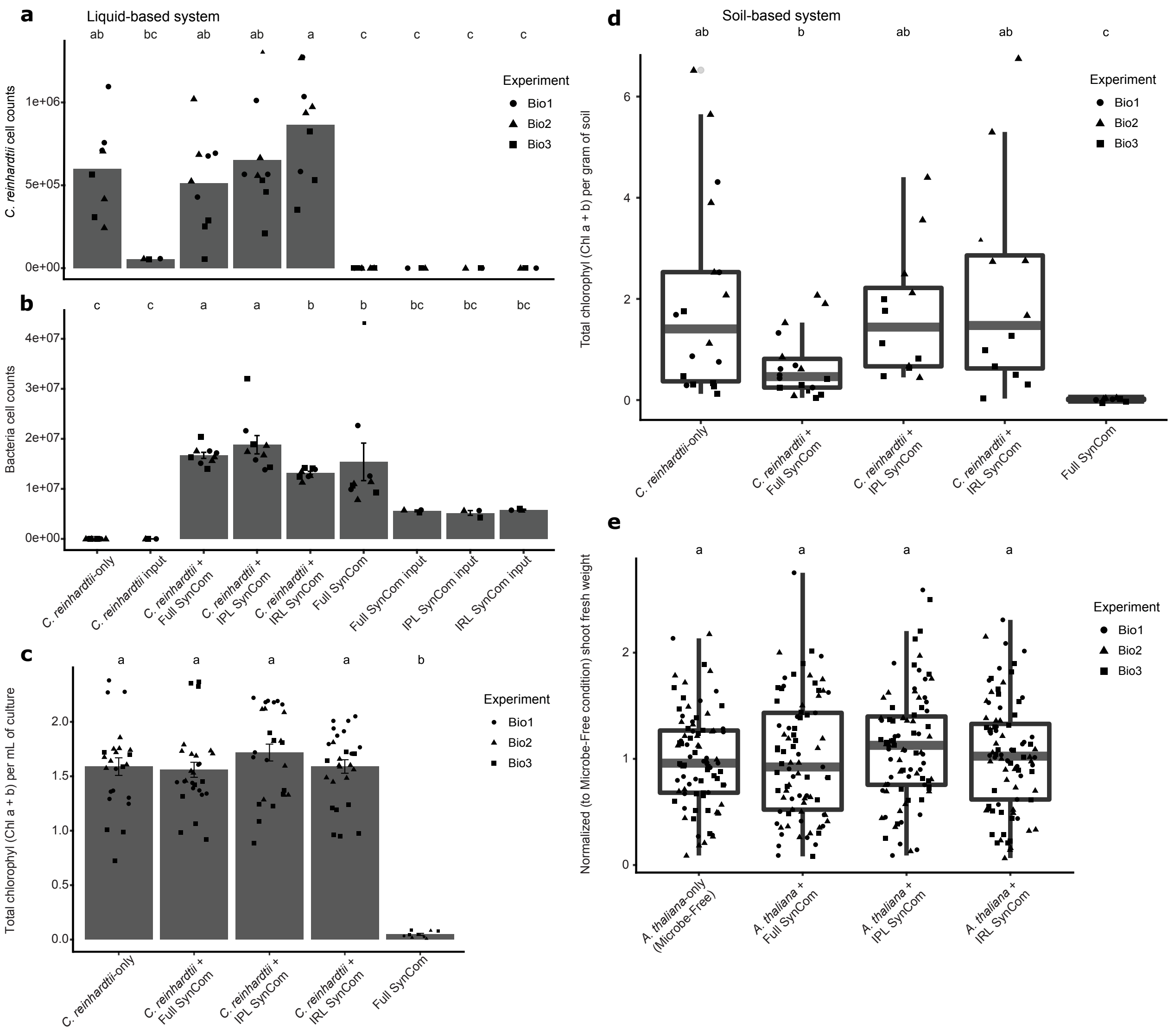

Supplementary Figure 7 | Bacterial and host growth parameters in cross-inoculation experiments with IPL and IRL bacterial SynComs.

(**a**) *Cr* cell counts after 7 days of co-incubation with different bacterial SynCom combinations in liquid system (n=56). (**b**) Cell counts of different bacterial SynCom combinations after 7 days of co-incubation with or without *Cr* in a liquid system (n=56). (**c**) *Cr* total chlorophyll content after 7 days of co-incubation in the liquid system (n=114). Error bars (**a-c**) represent the standard deviation of the data. (**d**) *Cr* total chlorophyll content after 4 weeks of co-incubation in the soil-based system (n=69). (**e**) *At* normalized shoot fresh wait after 4 weeks of co-incubation in the soil-based system (n=347). Letters indicate significant differences between condition within one culture collection (Kruskal-Wallis, followed by a Dunn's test *post hoc* and Bonferroni correction). Complements experiment G (**Supplementary Fig. 1g** and **Supplementary Table 1**).

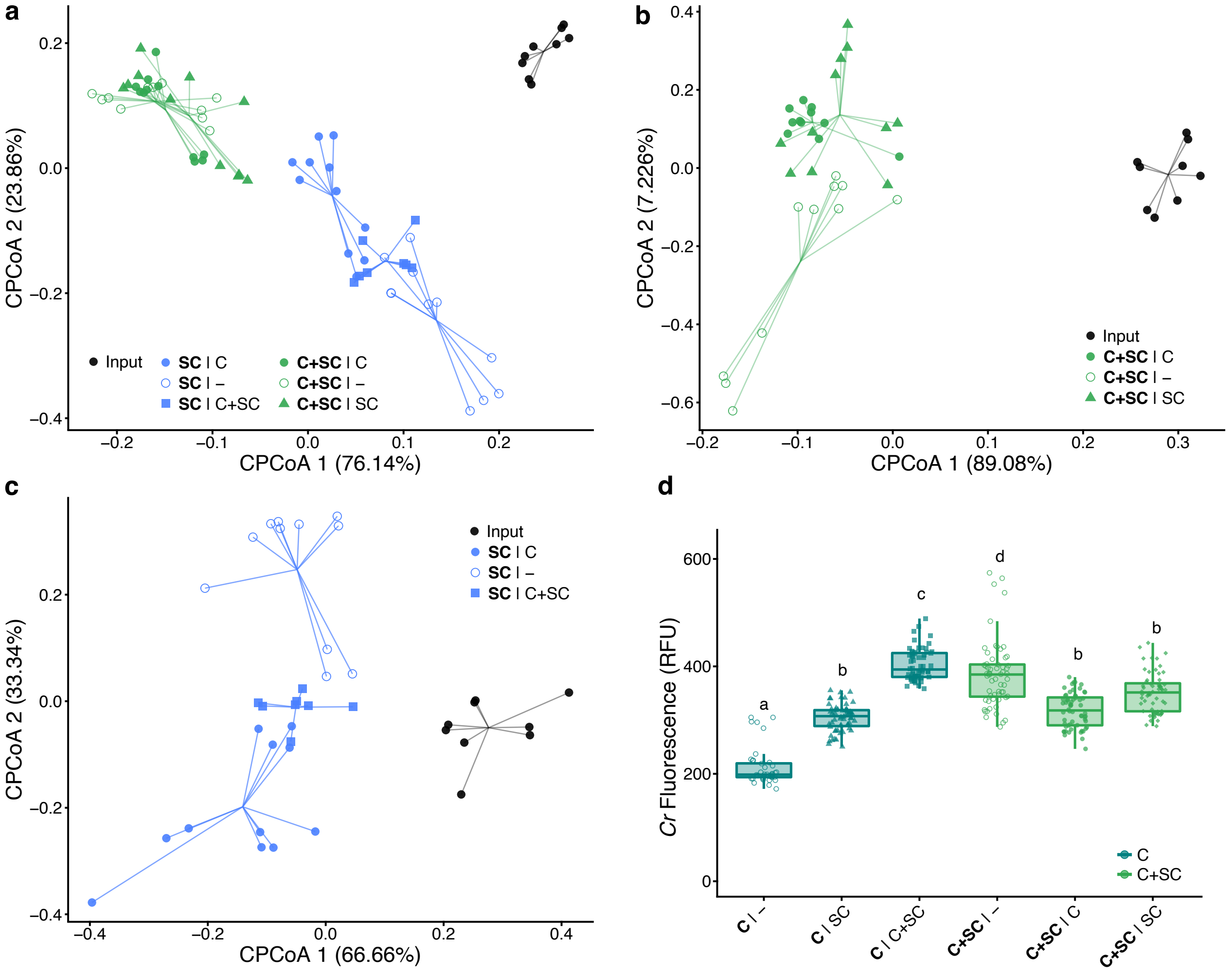

Supplementary Figure 8 | Effect of physical proximity in SynCom community structure and *Cr* growth (split bottle system).

Strain-level beta-diversity analyses of Bray-Curtis dissimilarities of bacterial SynComs (SC, n=30), and synthetic phycospheres (SC+C, n=33), grown in a split gnotobiotic system (split bottle system). Constrained PCoA is shown for all samples (20.6% of variance; P<0.001, **a**), or a subset of samples, depending on the content of the vessel (22.7-39.7% of variance; P<0.001, **b-c**). (**d**) *Cr* growth after 7 days of co-incubation with different compartment combinations, measured as relative chlorophyll fluorescence (RFU). Boxplots are color-coded depending on whether *Cr* was incubated alone in a given compartment (n=360, dark green) or in combination with SC (n=360, light green). Corresponds to experiment H (**Supplementary Fig. 1h** and **Supplementary Table 1**).

Supplementary Table 1 | List of the main experiments and summary of their conditions.

ID	System	Bacterial community	Media	Host	Compartments harvested	Sampling times	*n*	Analysis
**A**	Natural soil (CAS) in the greenhouse	Natural soil microbial community	Natural soil	*Cr* (CC1690)	Phycosphere	Day 36	24	*16S* rRNA
				At (Col-0)	Root		18	
					Rhizosphere		32	
				Uninoculated / unplanted	Soil	Day 0, 36	24	
**B**	Natural soil (CAS) in growth chambers	Natural soil microbial community	Natural soil	Native CAS microalgae	Soil surface (light / dark)	Week 7	17 / 18	*16S* / *18S* rRNA, chlorophyll (*Cr*)
					Soil surface (dark)		16 / 17	
					Initial soil	Day 0	3 / 4	
**C**	Liquid flask	Soil bacterial extracts (CAS and Golm)	TP, B&D	*Cr* (CC1690)	*Cr* + Bacteria	Days 1, 4, 7, 11	158	*16S* rRNA, chlorophyll / cell counts (*Cr*)
					Bacteria + AP			
					Bacteria alone		112	
					Initial soil extracts	Day 0	12	
**D**	Liquid flask	Soil bacterial extracts (CAS)	SFM	*Cr* (CC1690)	Phycosphere	Days 14, 21, 35	18	*16S* rRNA
				MEL 1108 B			18	
				MEL 1030 B			18	
				MEL 1121 B			21	
				MEL 1126 B			20	
				Uninoculated	Bacteria alone		14	
**F**	Liquid flask	*Cr*-SPHERE SynCom	TP	*Cr* (CC1690)	*Cr* + SynCom	Days 1, 4, 7	27	*16S* rRNA, chlorophyll / cell counts (*Cr*)
					Axenic *Cr*		25	
		Axenic		Uninoculated	SynCom alone	Days 0, 1, 4, 7	9	
**G**	FlowPot	*Cr*- and *At*-SPHERE SynComs	Sterile soil	*Cr* (CC1690)	Phycosphere	Week 5	66	*16S* rRNA, chlorophyll, cell counts (*Cr*), fresh weight (*At*)
				At (Col-0)	Root		57	
				Unplanted	SynCom alone		26	
	Liquid flask	*Cr*- and *At*-SPHERE SynComs	TP	*Cr* (CC1690)	Phycosphere	Day 7	27	
				Uninoculated	SynCom alone	Days 0, 7	12	
**H**	Split system	*Cr*-SPHERE SynCom	TP	*Cr* (CC1690)	*Cr* + SynCom	Day 7	46	*16S* rRNA, chlorophyll / cell counts (*Cr*)
					Axenic *Cr*		37	
				Uninoculated	SynCom alone	Days 0, 7	11	

Supplementary Table 2 | Composition of SFM culture medium.

	Components	Final concentration	Stock solution	Addition per liter
1	HEPES	1 mM	238.10 g / L dH_2O	1 mL
2	$Ca(NO_3)2$ x 4 H_2O	0.21 mM	100.00 g / L dH_2O	0.5 mL
3	$MgSO_4$ x 7 H_2O	0.203 mM	20.00 g / L dH_2O	2.5 mL
4	$(NH_4)_2HPO_4$	87.8 µm	20.00 g / L dH_2O	0.58 mL
5	K_2HPO_4 x 3 H_2O +	13.2 µm	5.00 g / L dH_2O	0.6 mL
	Na_2CO_3	0.19 mM	32.00 g / L dH_2O	
6	Na_2NO_3	0.176 mM	50.00 g / L dH_2O	0.3 mL
7	H_3BO_3	16 µm	1.00 g / L dH_2O	1 mL

8	Vitamin solution			1 mL
	Vitamin B12	0.15 nM	0.20 mg / L dH_2O	
	Biotin	4.10 nM	1.00 mg / L dH_2O	
	Thiamine-HCl	0.30 µm	100.00 mg / L dH_2O	
	Niacinamide	0.80 µm	0.10 mg / L dH_2O	
	pH 7			

9	Trace metals			1 mL
9	*Trace metal solution preparation*			
	Na_2EDTA x 2 H_2O		4.36 g / L dH_2O	
	$FeCl_3$ x 6 H_2O		3.15 g / L dH_2O	
	Dissolve in 1000 ml dH2O, then add 1 ml of Primary Trace Metals each (see below). Primary Trace Metals are stored frozen as 1 mL aliquots.			
9	*Primary trace metals*			
	$CoCl_2$ x 6 H_2O		1.00 g / 100 mL dH_2O	
	$CuSO_4$ x 5 H_2O		0.25 g / 100 mL dH_2O	
	$MnCl_2$ x 4 H_2O		18.00 g / 100 mL dH_2O	
	Na_2MoO_4 x 2 H_2O		1.89 g / 100 mL dH_2O	
	$NiSO_4$ x 6 H_2O		0.27 g / 100 mL dH_2O	
	H_2SeO_3		0.13 g / 100 mL dH_2O	
	Na_3VO_4		0.184 g / 100 mL dH_2O	
	$ZnSO_4$ x 7 H_2O		2.20 g / 100 mL dH_2O	

pH is adjusted to pH 6.0 with HCl
