# Supplementary material for: Shared features and reciprocal complementation of the Chlamydomonas and Arabidopsis microbiota
Source: Nat Commun. 2022 Jan 20;13:406. doi: 10.1038/s41467-022-28055-8 (PMC8776852; doi:10.1038/s41467-022-28055-8)
Supplement: Supplementary file 3 — Description of Additional Supplementary Files [file 41467_2022_28055_MOESM3_ESM.pdf]

Description of Additional Supplementary Files

File Name: Supplementary Data 1
Description: Abundant community members in *Cr* phycosphere and *At* root samples.

File Name: Supplementary Data 2
Description: Recovery of abundant phycosphere bacteria in the IPL culture collection.

File Name: Supplementary Data 3
Description: Metadata and assembly statistics of isolates constituting the *Cr*-SPHERE core collection.

File Name: Supplementary Data 4
Description: Strain and taxonomic composition of SynComs used in this study.
