# Supplementary material for: Shared features and reciprocal complementation of the Chlamydomonas and Arabidopsis microbiota
Source: Nat Commun. 2022 Jan 20;13:406. doi: 10.1038/s41467-022-28055-8 (PMC8776852; doi:10.1038/s41467-022-28055-8)
Supplement: Supplementary file 8 — Reporting Summary [file 41467_2022_28055_MOESM8_ESM.pdf]

nature portfolio

Corresponding author(s): Ruben Garrido-Oter
Last updated by author(s): Dec 3, 2021

Reporting Summary

Nature Portfolio wishes to improve the reproducibility of the work that we publish. This form provides structure for consistency and transparency in reporting. For further information on Nature Portfolio policies, see our Editorial Policies and the Editorial Policy Checklist.

Statistics

For all statistical analyses, confirm that the following items are present in the figure legend, table legend, main text, or Methods section.

n/a	Confirmed	
☐	☒	The exact sample size (*n*) for each experimental group/condition, given as a discrete number and unit of measurement
☐	☒	A statement on whether measurements were taken from distinct samples or whether the same sample was measured repeatedly
☐	☒	The statistical test(s) used AND whether they are one- or two-sided<br>*Only common tests should be described solely by name; describe more complex techniques in the Methods section.*
☒	☐	A description of all covariates tested
☐	☒	A description of any assumptions or corrections, such as tests of normality and adjustment for multiple comparisons
☐	☒	A full description of the statistical parameters including central tendency (e.g. means) or other basic estimates (e.g. regression coefficient) AND variation (e.g. standard deviation) or associated estimates of uncertainty (e.g. confidence intervals)
☐	☒	For null hypothesis testing, the test statistic (e.g. *F*, *t*, *r*) with confidence intervals, effect sizes, degrees of freedom and *P* value noted<br>*Give P values as exact values whenever suitable.*
☒	☐	For Bayesian analysis, information on the choice of priors and Markov chain Monte Carlo settings
☐	☒	For hierarchical and complex designs, identification of the appropriate level for tests and full reporting of outcomes
☒	☐	Estimates of effect sizes (e.g. Cohen's *d*, Pearson's *r*), indicating how they were calculated

Our web collection on statistics for biologists contains articles on many of the points above.

Software and code

Policy information about availability of computer code

Data collection

Fluorescence and absorbance measurements were obtained using Tecan i-control (v2.0.10.0); cell counts were obtained using Beckmain Coulter Multisizer (v4.03).

Data analysis

Data analyses were performed using custom scripts, available at https://github.com/garridoo/crsphere

The following software was also used for data analysis:

Trimmomatic v0.32
IDBA v1.1.3
Prokka v.1.12-beta
Clustal Omega v1.2.0
FastTree v2.1.3
R statistical environment v4.0.3.
Vegan, R package v2.5-6
ggplot2, R package v3.3.2
USEARCH v8.0.1517_i86linux64
FastANI v1.32
A5 pipeline a5_miseq_linux_20160825
DADA2 v1.12.1
CheckM v1.1.2
Rbec v1.0.0

For manuscripts utilizing custom algorithms or software that are central to the research but not yet described in published literature, software must be made available to editors and reviewers. We strongly encourage code deposition in a community repository (e.g. GitHub). See the Nature Portfolio guidelines for submitting code & software for further information.

Data

Policy information about availability of data

All manuscripts must include a data availability statement. This statement should provide the following information, where applicable:

- Accession codes, unique identifiers, or web links for publicly available datasets
- A description of any restrictions on data availability
- For clinical datasets or third party data, please ensure that the statement adheres to our policy

Raw sequencing data has been deposited into the European Nucleotide Archive (ENA) under the accession number PRJEB43117 [https://www.ebi.ac.uk/ena/browser/view/PRJEB43117]. The scripts used for the computational analyses described in this study are available at http://www.github.com/garridoo/crsphere, to ensure replicability and reproducibility of these results. Source data are also provided with this paper. In addition, sequencing data, intermediate results, and metadata tables can be downloaded as a bundle from: http://www.at-sphere.com/cr.tar.gz

Field-specific reporting

Please select the one below that is the best fit for your research. If you are not sure, read the appropriate sections before making your selection.

☒ Life sciences ☐ Behavioural & social sciences ☐ Ecological, evolutionary & environmental sciences

For a reference copy of the document with all sections, see nature.com/documents/nr-reporting-summary-flat.pdf

Life sciences study design

All studies must disclose on these points even when the disclosure is negative.

Sample size	Sample sizes were chosen based on logistical and experimental constrains. In every case, a minimum of three biological and three technical replicates for each experiment were included. All experiments were reliably reproduced and the number of samples chosen was sufficient to support meaningful conclusions.
Data exclusions	No data exclusions were performed.
Replication	Multiple biological replicates were used as indicated in the Methods section and all experiments were reliably reproduced.
Randomization	Inoculation, harvesting, and processing of samples was performed randomly for each biological replicate. This work did not involve experimental groups.
Blinding	This work does not involve participant or experimental groups, therefore blinding was not necessary.

Reporting for specific materials, systems and methods

We require information from authors about some types of materials, experimental systems and methods used in many studies. Here, indicate whether each material, system or method listed is relevant to your study. If you are not sure if a list item applies to your research, read the appropriate section before selecting a response.

Materials & experimental systems

n/a	Involved in the study	
☒	☐	Antibodies
☒	☐	Eukaryotic cell lines
☒	☐	Palaeontology and archaeology
☒	☐	Animals and other organisms
☒	☐	Human research participants
☒	☐	Clinical data
☒	☐	Dual use research of concern

Methods

n/a	Involved in the study	
☒	☐	ChIP-seq
☒	☐	Flow cytometry
☒	☐	MRI-based neuroimaging
